# Supplementary material for: The balance stabilising benefit of social touch: Influence of an individual’s age and the partner’s relative body characteristics
Source: PLoS One. 2025 Jun 5;20(6):e0314946. doi: 10.1371/journal.pone.0314946 (PMC12140250; doi:10.1371/journal.pone.0314946)
Supplement: S1 Table — EO: Eyes open, EC: Eyes closed; IPT: interpersonal touch. (DOCX) [file pone.0314946.s001.docx]

**Supporting information and materials**

**S1 Table.** **Descriptive statistics of the entire participant sample: individual characteristics.** EO: Eyes open, EC: Eyes closed; IPT: interpersonal touch. BCa95%CI: Bias corrected and accelerated 95% confidence interval.

| Parameters |  |
| --- | --- |
| Sex: f/m | N=70 (48.6%) / N=74 (51.4%) |
| Age-related motor experience (y) | M=20.65, BCa95%CI [18.32 22.97]  SD=15.25, BCa95%CI [13.89 16.43]  Min=4, Max=63 |
| Height (m) | M=1.54, BCa95%CI [1.51 1.57]  SD=0.20, BCa95%CI [0.18 0.21]  Min=1.12, Max=1.94 |
| Weight (kg) | M=49.87, BCa95%CI [46.10 53.55]  SD=24.61, BCa95%CI [22.31 26.91]  Min=12, Max=123 |
| BMI (kg/m2) | M=19.41, BCa95%CI [18.56 20.31]  SD=5.49, BCa95%CI [4.97 5.99]  Min=9.3 , Max=35.5 |
| Balancing skill, EO - no IPT (SD dCoP; mm/s2) | M=59.07, BCa95%CI [54.76 63.98]  SD=28.62, BCa95%CI [23.55 33.22]  Min=19.60, Max=167.16 |
| Balancing skill, EC - no IPT (SD dCoP; mm/s2) | M=211.74, BCa95%CI [192.25 230.48]  SD=111.49, BCa95%CI [99.85 122.01]  Min=51.47, Max=513.642 |
| Pairing: Same sex / different sex | N=76 (52.8%), N=68 (47.2%) |
