# Supplementary material for: The balance stabilising benefit of social touch: Influence of an individual’s age and the partner’s relative body characteristics
Source: PLoS One. 2025 Jun 5;20(6):e0314946. doi: 10.1371/journal.pone.0314946 (PMC12140250; doi:10.1371/journal.pone.0314946)
Supplement: S2 Table — EO: Eyes open, EC: Eyes closed; IPT: interpersonal touch. (DOCX) [file pone.0314946.s002.docx]

**Supporting information and materials**

**S2 Table.** **Descriptive statistics of the entire participant sample: relative interindividual differences in characteristics between interaction partners.** EO: Eyes open, EC: Eyes closed; IPT: interpersonal touch. BCa95%CI: Bias corrected and accelerated 95% confidence interval.

| Difference in age-related motor experience (y) | M=0.30, BCa95%CI [-2.84 3.48]  SD=19.30, BCa95%CI [17.57 20.87]  Min=-40, Max=40 |
| --- | --- |
| Height difference (m) | M=0.00, BCa95%CI [-0.04 0.04]  SD=0.23, BCa95%CI [0.20 0.25]  Min=-67, Max=67 |
| Weight difference (kg) | M=0.00, BCa95%CI [-4.73 5.16]  SD=31.07, BCa95%CI [27.58 34.35]  Min=-78, Max=78 |
| BMI difference (kg/m2) | M=0.00, BCa95%CI [-1.16 1.21]  SD=7.32, BCa95%CI [6.59 7.99]  Min=-16.20, Max=16.20 |
| Difference in balancing skills, EO (mm/s2) | M=0.16, BCa95%CI [0.04 0.29]  SD=0.71, BCa95%CI [0.51 0.91]  Min=-0.81, Max=4.33 |
| Relative benefit of IPT, EO (relative sway change due to IPT EO;mm/s2)) | M=-13.58, BCa95%CI [-18.41 -9.00]  SD=27.83, BCa95%CI [22.63 32.77]  Min=-111.18, Max=94.87 |
| Percentage benefit of IPTm EO (percentage sway change due to IPT, EO (%)) | M=-17.01, BCa95%CI [-22.32 -10.93]  SD=34.14, BCa95%CI [22.61 41.79]  Min=-79.89, Max=153.31 |
| Difference in balancing skills, EC (mm/s2) | M=0.26, BCa95%CI [0.12 0.42]  SD=0.89, BCa95%CI [0.50 0.91]  Min=-0.81, Max=4.33 |
| Relative benefit of IPT, EC (relative sway change due to IPT, EC (mm/s2)) | M=-75.74, BCa95%CI [-94.93 -56.53]  SD=112.71, BCa95%CI [99.96 124.24]  Min=-382.65, Max=188.40 |
| Percentage benefit of IPT, EC (sway change due to IPT, EC (%)) | M=-24.25, BCa95%CI [-32.17 -15.53]  SD=52.90, BCa95%CI [42.47 62.39]  Min=-85.27, Max=216.08 |
