# Supplementary material for: The balance stabilising benefit of social touch: Influence of an individual’s age and the partner’s relative body characteristics
Source: PLoS One. 2025 Jun 5;20(6):e0314946. doi: 10.1371/journal.pone.0314946 (PMC12140250; doi:10.1371/journal.pone.0314946)
Supplement: S3 Table — Significant cluster differences are indicated by a star and statistics are shown in the last column (equal variances not assumed). EO: Eyes open, EC: Eyes closed; IPT: interpersonal touch. (DOCX) [file pone.0314946.s003.docx]

**Supporting information and materials**

**S3 Table.** Participant characteristics for the two performance clusters based on the Eyes open condition. Significant cluster differences are indicated by a star and statistics are shown in the last column (equal variances not assumed). EO: Eyes open, EC: Eyes closed; IPT: interpersonal touch. BCa95%CI: Bias corrected and accelerated 95% confidence interval.

|  | Cluster 1 (N=52) | Cluster 2 (N=92) | absolute Cluster differences (boot-strapped Chi2-test and t-test; N=1000; seed=2021, independent samples effect size) |
| --- | --- | --- | --- |
| Sex: f/m | N=21 (40.4%) / N=31 (59.6%) | N=49 (53.3%) / N=43 (46.7%) | Chi2=0.138, Phi=-0.124 |
| Age-related motor experience (y) | M=37.35, BCa95% [34.10 40.33]  SD=11.49, BCa95% [9.37 13.10]  Min=8, Max=63 | M=11.21, BCa95% [10.22 12.38]  SD=6.49, BCa95% [3.56 8.59]  Min=4, Max=50 | **Mdiff=26.14, p<0.001**  **BCa95%CI [22.90 29.42]**  **d=3.03 95%CI [2.54 3.52]** |
| Height (m) | M=1.75, BCa95% [1.71 1.78]  SD=0.10, BCa95% [0.08 0.12]  Min=1.42, Max=1.94 | M=1.43, BCa95% [1.41 1.46]  SD=0.14, BCa95% [0.12 0.15]  Min=1.12, Max=1.75 | **Mdiff=0.32, p<0.001**  **BCa95%CI [0.28 0.36]**  **d=2.50 95%CI [2.05 2.95]** |
| Weight (kg) | M=77.06, BCa95% [73.13 81.06]  SD=15.15, BCa95% [12.45 17.46]  Min=47, Max=123 | M=34.50, BCa95% [32.30 36.96]  SD=12.73, BCa95% [11.25 13.82]  Min=12, Max=64 | **Mdiff=42.56, p<0.001**  **BCa95%CI [37.79 47.79]**  **d=3.12 95%CI [2.62 3.61]** |
| BMI (kg/m2) | M=25.10, BCa95% [24.26 25.85]  SD=3.42, BCa95% [2.82 3.89]  Min=18.9, Max=35.5 | M=16.20, BCa95% [15.58 16.84]  SD=3.43, BCa95% [3.07 3.75]  Min=9.3, Max=24.2 | **Mdiff=8.91, p<0.001**  **BCa95%CI [7.72 10.10]**  **d=2.60 95%CI [2.14 3.05]** |
| Variability in balancing performance, EO - no IPT (SD dCoP (mm/s2)) | M=50.64, BCa95% [45.67 56.47]  SD=20.55, BCa95% [14.67 25.14]  Min=19.60, Max=122.61 | M=63.83, BCa95% [58.20 69.66]  SD=31.41, BCa95% [25.18 36.28]  Min=25.36, Max=167.16 | **Mdiff=13.19, p<0.005**  **BCa95%CI [-21.50 -4.55]**  **d=-0.47 95%CI [-0.82 -0.13]** |
| Pairing: Same sex / different sex | N=29 (55.8%) / N=23 (44.2%) | N=47 (51.1%) / N=45 (48.9%) | Chi2=0.589, Phi=-0.045 |
| Difference in age-related motor experience (y) | M=15.83, BCa95% [11.80 19.99]  SD=15.50, BCa95% [13.66 16.81]  Min=-16, Max=38 | M=-8.48, BCa95% [-11.37 -5.45]  SD=15.34, BCa95% [13.50 16.89]  Min=-40, Max=40 | **Mdiff=24.31, p<0.001**  **BCa95%CI [19.19 29.144]**  **d=1.58 95%CI [1.19 1.96]** |
| Height difference (m) | M=0.15, BCa95% [0.10 0.20]  SD=0.20, BCa95% [0.16 0.22]  Min=-0.20, Max=0.67 | M=-0.09, BCa95% [-0.12 -0.05]  SD=0.19, BCa95% [0.17 0.22]  Min=-0.67, Max=0.33 | **Mdiff=0.24, p<0.001**  **BCa95%CI [0.18 0.30]**  **d=1.22 95%CI [0.86 1.59]** |
| Weight difference (kg) | M=22.85, BCa95% [15.49 30.09]  SD=29.26, BCa95% [24.06 33.50]  Min=-62, Max=78 | M=-12.91, BCa95% [-17.73 -8.09]  SD=23.88, BCa95% [20.64 26.53]  Min=-78, Max=29 | **Mdiff=35.76, p<0.001**  **BCa95%CI [26.61 44.55]**  **d=1.38 95%CI [1.00 1.75]** |
| BMI difference (kg/m2) | M=5.24, BCa95% [3.42 6.80]  SD=6.84, BCa95% [5.60 7.87]  Min=-16.20, Max=16.20 | M=-2.96, BCa95% [-4.12 -1.74]  SD=5.78, BCa95% [5.10 6.32]  Min=-16.00, Max=8.90 | **Mdiff=8.20, p<0.001**  **BCa95%CI [5.96 10.33]**  **d=1.33 95%CI [0.95 1.70]** |
| Difference in balancing performance, EO - no IPT (mm/s2) | M=-0.06, BCa95% [-0.15 0.03]  SD=0.36, BCa95% [0.31 0.40]  Min=-0.66, Max=0.67 | M=0.27, BCa95% [0.12 0.43]  SD=0.82, BCa95% [0.60 1.02]  Min=-0.81, Max=4.33 | **Mdiff=0.33, p=0.005**  **BCa95%CI [-0.53 -0.15]**  **d=-0.48 95%CI [-0.82 -0.13]** |
| Benefit of IPT, EO (relative change due to IPT, EO (mm/s2)) | M=-7.59, BCa95% [-14.13 -0.15]  SD=25.44, BCa95% [15.59 32.74]  Min=-72.33, Max=94.87 | M=-16.97, BCa95% [-22.58 -11.10]  SD=28.67, BCa95% [23.85 32.90]  Min=-11.18, Max=64.86 | **Mdiff=9.38, p=0.046**  **BCa95%CI [0.90 18.19]**  **d=0.34 95%CI [-0.00 0.68]** |
| Benefit of IPT, EO (percentage change due to IPT EO (%)) | M=-10.79, BCa95% [-20.36 1.22]  SD=40.05, BCa95% [21.94 53.46]  Min=-62.99, Max=153.31 | M=-20.53, BCa95% [-25.99 -14.56]  SD=29.97, BCa95% [25.47 33.65]  Min=-79.89, Max=74.73 | Mdiff=9.73, p=0.14  BCa95%CI [-1.61 21.47]  d=0.29 95%CI [-0.06 0.63] |

Difference in balancing skill: (balancing skill self - balancing skill other) / (balancing skill other);
Difference in benefit of IPT (%): (balance performance with IPT - balance performance wo IPT) / balance performance wo IPT)*100
