# Supplementary material for: The balance stabilising benefit of social touch: Influence of an individual’s age and the partner’s relative body characteristics
Source: PLoS One. 2025 Jun 5;20(6):e0314946. doi: 10.1371/journal.pone.0314946 (PMC12140250; doi:10.1371/journal.pone.0314946)
Supplement: S4 Table — Significant cluster differences are indicated by a star and statistics are shown in the last column (equal variances not assumed). EO: Eyes open, EC: Eyes closed; IPT: interpersonal touch. (DOCX) [file pone.0314946.s004.docx]

**Supporting information and materials**

**S4 Table.** Participant characteristics for the two performance clusters based on Eyes closed condition. Significant cluster differences are indicated by a star and statistics are shown in the last column (equal variances not assumed). EO: Eyes open, EC: Eyes closed; IPT: interpersonal touch. BCa95%CI: Bias corrected and accelerated 95% confidence interval.

|  | Cluster 1 (N=57) | Cluster 2 (N=87) | absolute Cluster differences (boot-strapped Chi2 and t-tests; N=1000; seed=2021) |
| --- | --- | --- | --- |
| Sex: f/m | N=24 (42.1%) / N=33 (57.9%) | N=46 (52.9%) / N=41 (47.1%) | p=0.206, Phi=-0.105 |
| Age-related motor experience (y) | M=36.74, BCa95% [33.72 39.75]  SD=11.91, BCa95% [10.06 13.36]  Min=8, Max=63 | M=10.10, BCa95% [9.49, 10.76]  SD=3.21, BCa95% [2.26 4.03]  Min=4, Max=27 | **Mdiff=26.63, p<0.001**  **BCa95%CI [23.40 29.78]**  **d=3.38 95%CI [2.86 3.89]** |
| Height (m) | M=1.74, BCa95% [1.71 1.77]  SD=0.10, BCa95% [0.08 0.11]  Min=1.42, Max=1.94 | M=1.42, BCa95% [1.39 1.45]  SD=0.13, BCa95% [0.11 0.14]  Min=1.12, Max=1.75 | **Mdiff=0.32, p<0.001**  **BCa95%CI [0.29 0.36]**  **d=2.73 95%CI [2.27 3.19]** |
| Weight (kg) | M=75.60, BCa95% [72.14 79.18]  SD=15.23, BCa95% [12.42 17.59]  Min=47, Max=123 | M=33.01, BCa95% [30.41 35.69]  SD=11.40, BCa95% [10.20 12.61]  Min=12, Max=64 | **Mdiff=42.59, p<0.001**  **BCa95%CI [38.02 47.23]**  **d=3.27 95%CI [2.76 3.77]** |
| BMI (kg/m2) | M=24.80, BCa95% [23.99 25.67]  SD=3.42, BCa95% [2.79 3.98]  Min=18.9, Max=35.5 | M=15.88, BCa95% [15.11 16.66]  SD=3.25, BCa95% [2.89 3.60]  Min=9.3, Max=24.2 | **Mdiff=8.92, p<0.001**  **BCa95%CI [7.86 9.99]**  **d=2.69 95%CI [2.23 3.14]** |
| Variability in balancing performance, EC (SD dCoP; mm/s2) | M=213.99, BCa95% [185.04 242.53]  SD=120.05, BCa95% [102.96 132.83]  Min=61.62, Max=487.91 | M=201.26, BCa95% [188.84 232.83]  SD=106.20, BCa95% [90.95 119.97]  Min=51.47 513.64 | Mdiff=3.73, p=0.84  BCa95%CI [-35.27 42.79]  d=0.03 95%CI [-0.30 0.37] |
| Pairing: Same sex / different sex | N=32 (56.1%) / 25 (43.9%) | N=44 (50.6%) / 43 (49.4%) | p=0.513, Phi=-0.055 |
| Difference in age-related motor experience (y) | M=15.75, BCa95% [12.23 19.04]  SD=15.55, BCa95% [13.89 16.86]  Min=-16, Max=40 | M=-9.83, BCa95% [-13.02 -7.01]  SD=14.15, BCa95% [12.55 15.40]  Min=-40, Max=6 | **Mdiff=25.58, p<0.001**  **BCa95%CI [20.92 30.52]**  **d=1.74 95%CI [1.35 2.13]** |
| Height difference (m) | M=0.15, BCa95% [0.10 0.19]  SD=0.19, BCa95% [0.16 0.22]  Min=-0.20, Max=0.67 | M=-0.10, BCa95% [-0.13 -0.06]  SD=0.19, BCa95% [0.164 0.22]  Min=-0.67, Max=0.33 | **Mdiff=0.24, p<0.001**  **BCa95%CI [0.19 0.31]**  **d=1.25 95%CI [0.89 1.62]** |
| Weight difference (kg) | M=21.49, BCa95% [14.27 28.29]  SD=28.80, BCa95 [24.09 33.32]  Min=-62, Max=78 | M=-14.08, BCa95% [-18.90 -9.19]  SD=23.63, BCa95% [19.79 26.82]  Min=-78, Max=29 | **Mdiff=35.57, p<0.001**  **BCa95%CI [27.46 43.92]**  **d=1.38 95%CI [1.01 1.75]** |
| BMI difference (kg/m2) | M=4.84, BCa95% [3.05 6.46]  SD=6.96, BCa9% [5.73 8.12]  Min=-16.20, Max=16.20 | M=-3.17, BCa95% [-4.3 -1.99]  SD=5.64, BCa95% [4.93 6.31]  Min=-16.00, Max=8.90 | **Mdiff=8.01, p<0.001**  **BCa95%CI [5.93 10.01]**  **d=1.29 95%CI [0.93 1.66]** |
| Difference in balancing skill, EC (mm/s2) | M=0.29, BCa95% [0.07 0.52]  SD=0.90, BCa9% [0.71 1.05]  Min=-0.78, Max=3.15 | M=0.24, BCa95% [0.07 0.45]  SD=0.88, BCa95% [0.70 1.05]  Min=-0.76, Max=3.60 | Mdiff=0.05, p=0.68  BCa95%CI [-33.60 49.92]  d=0.05 95%CI [-0.28 0.39] |
| Benefit of IPT, EC (relative change due to IPT, EC (mm/s2)) | M=-70.57, BCa95% [-102.24 -35.51]  SD=125.13, BCa95% [106.78 141.30]  Min=-339.27, Max=188.40 | M=-79.12, BCa95%[-100.62 -58.88]  SD=104.40, BCa95% [82.57 119.99]  Min=-382.64, Max=163.76 | Mdiff=8.54, p=0.66  BCa95%CI [-30.70 45.32]  d=0.08 95%CI [-0.26 0.41] |
| Benefit of IPT (percentage sway change due to IPT, EC (%)) | M=-15.40, BCa95% [-31.20 3.64]  SD=67.57, BCa95% [46.10 84.80]  Min=-85.27, Max=216.08 | M=-30.05, BCa95% [-38.75 -22.31]  SD=39.94, BCa95% [33.03 45.74]  Min=-84.83, Max=82.96 | Mdiff=14.65, p=0.17  BCa95%CI [-4.50 34.88]  d=0.28 95%CI [-0.06 0.61] |

Proportional difference in balancing skill: (balancing skill self - balancing skill other) / (balancing skill other);
Percentage benefit of IPT: (balance performance with IPT - balance performance wo IPT) / balance performance wo IPT)*100
