# Supplementary figures and images for: The balance stabilising benefit of social touch: Influence of an individual’s age and the partner’s relative body characteristics
Source: PLoS One. 2025 Jun 5;20(6):e0314946. doi: 10.1371/journal.pone.0314946 (PMC12140250; doi:10.1371/journal.pone.0314946)

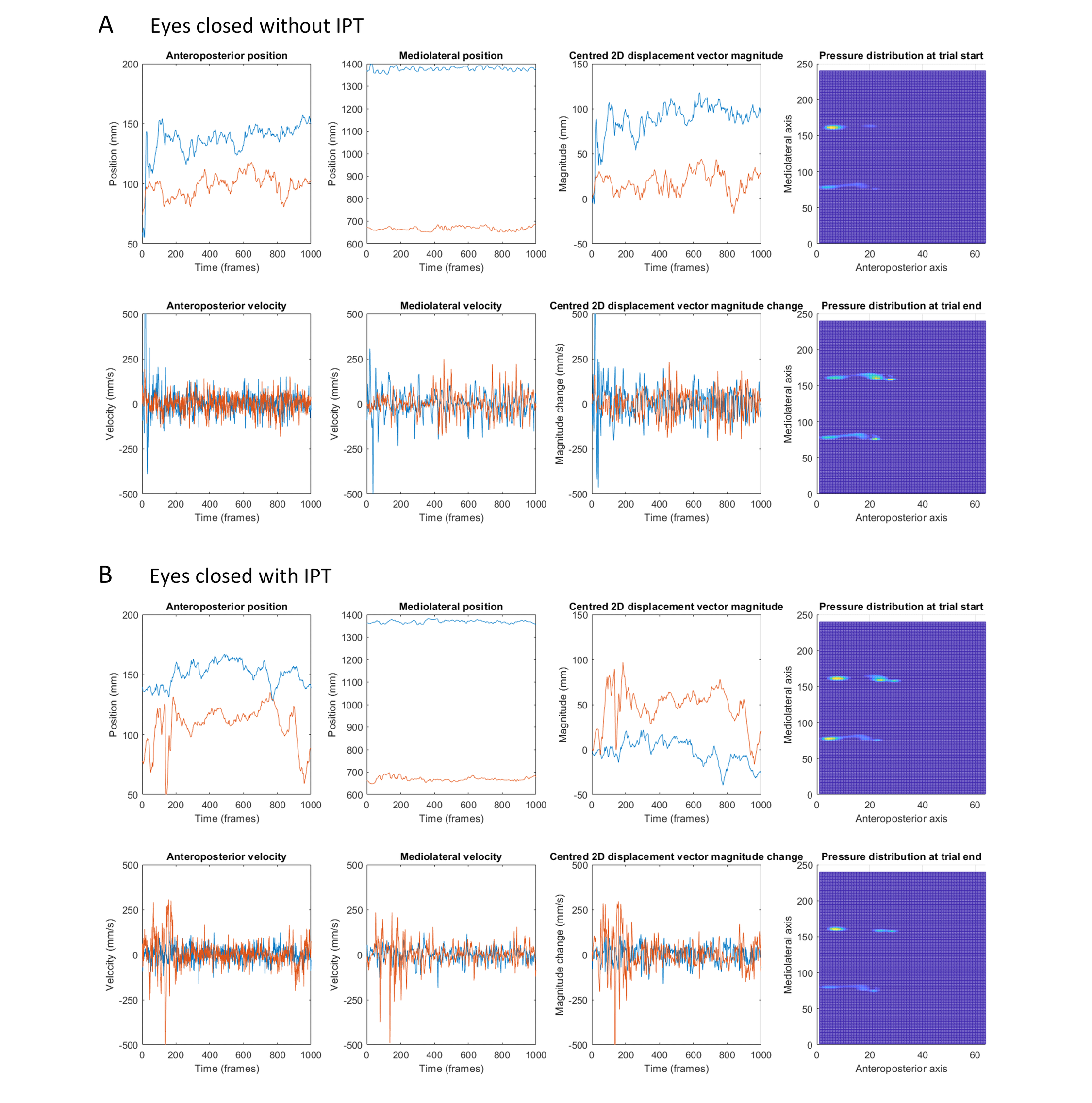

Supplement: S1 Fig — Illustrative data traces showing two individuals during side-by-side single-legged stance on a barometric platform. with (A) Eyes closed without interpersonal touch and with (B) Eyes closed and simultaneous interpersonal touch. The participant on the left of the pair is shown as a line in Blue, the participant on the right as a line in Orange. The 2D displacement vector magnitude is the resultant of the anteroposterior and mediolateral positions. IPT: interpersonal touch. (TIF) [file pone.0314946.s005.tif]

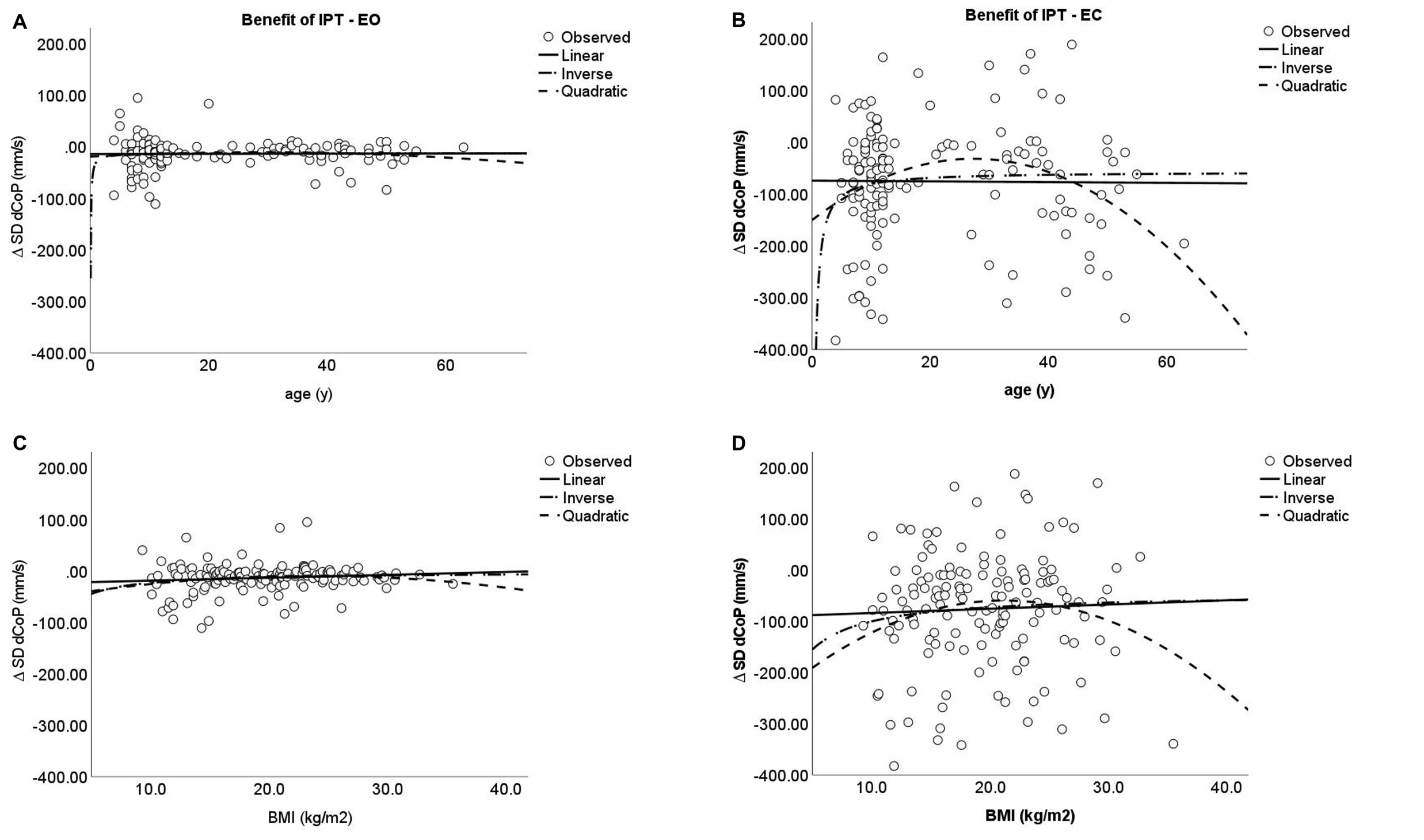

Supplement: S2 Fig — Curve fitting results for age (left) and BMI (right) with the benefit of IPT in Eyes open condition (EO; left) and Eyes closed condition (EC; right). (TIF) [file pone.0314946.s006.tif]

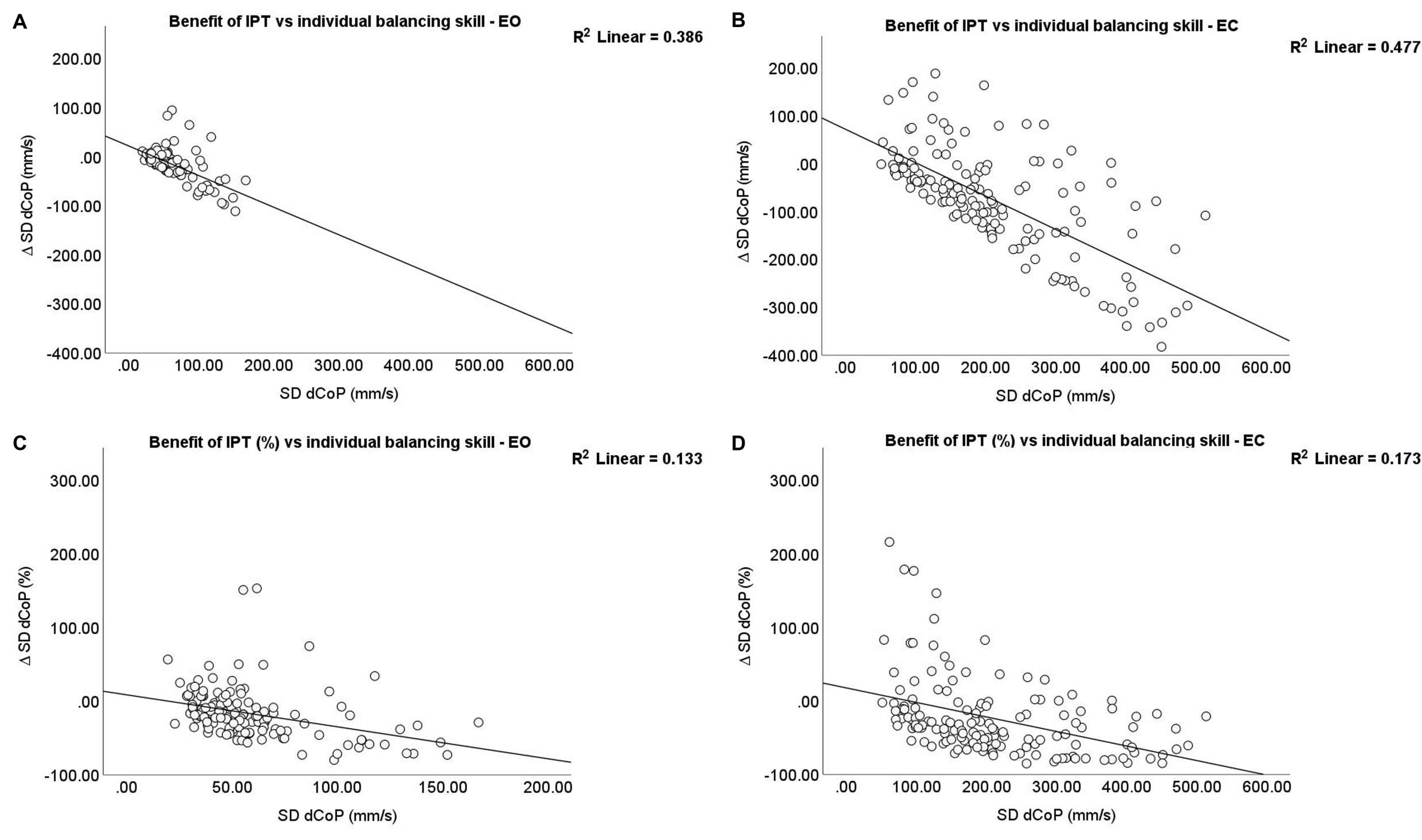

Supplement: S3 Fig — Scatterplot of the benefit of IPT (y-axis) in relation to an individual’s balancing skill (x-axis). A more negative delta SD dCoP (top) and percentage change in SD dCoP (bottom) indicate a greater sway reduction. EO: Eyes open, EC: Eyes closed; IPT: interpersonal touch. (TIF) [file pone.0314946.s007.tif]

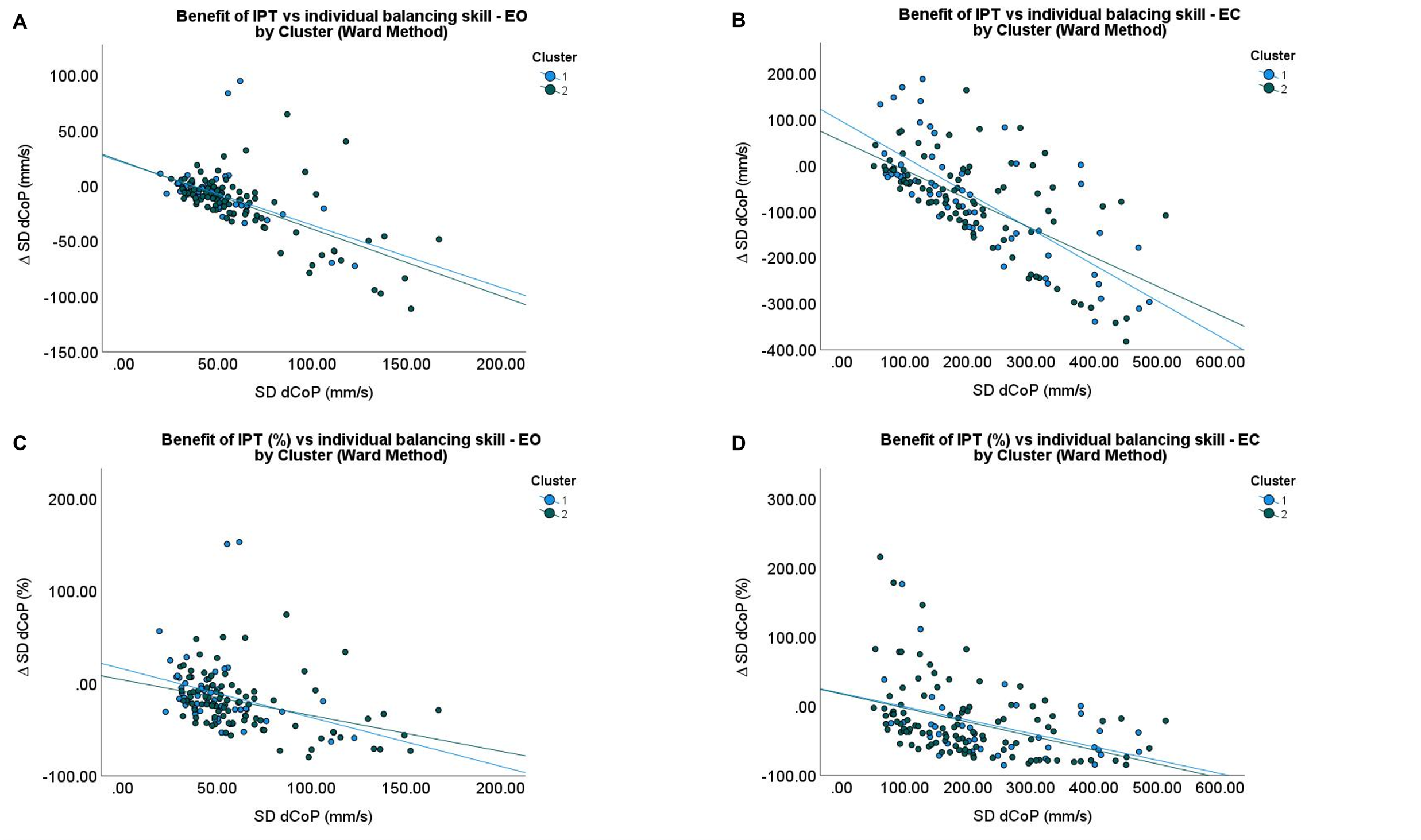

Supplement: S4 Fig — Scatterplot of an individual’s balancing skill and interindividual differences in balancing skills. with the benefit of IPT dependent on the personalised performance cluster assignment. (TIF) [file pone.0314946.s008.tif]

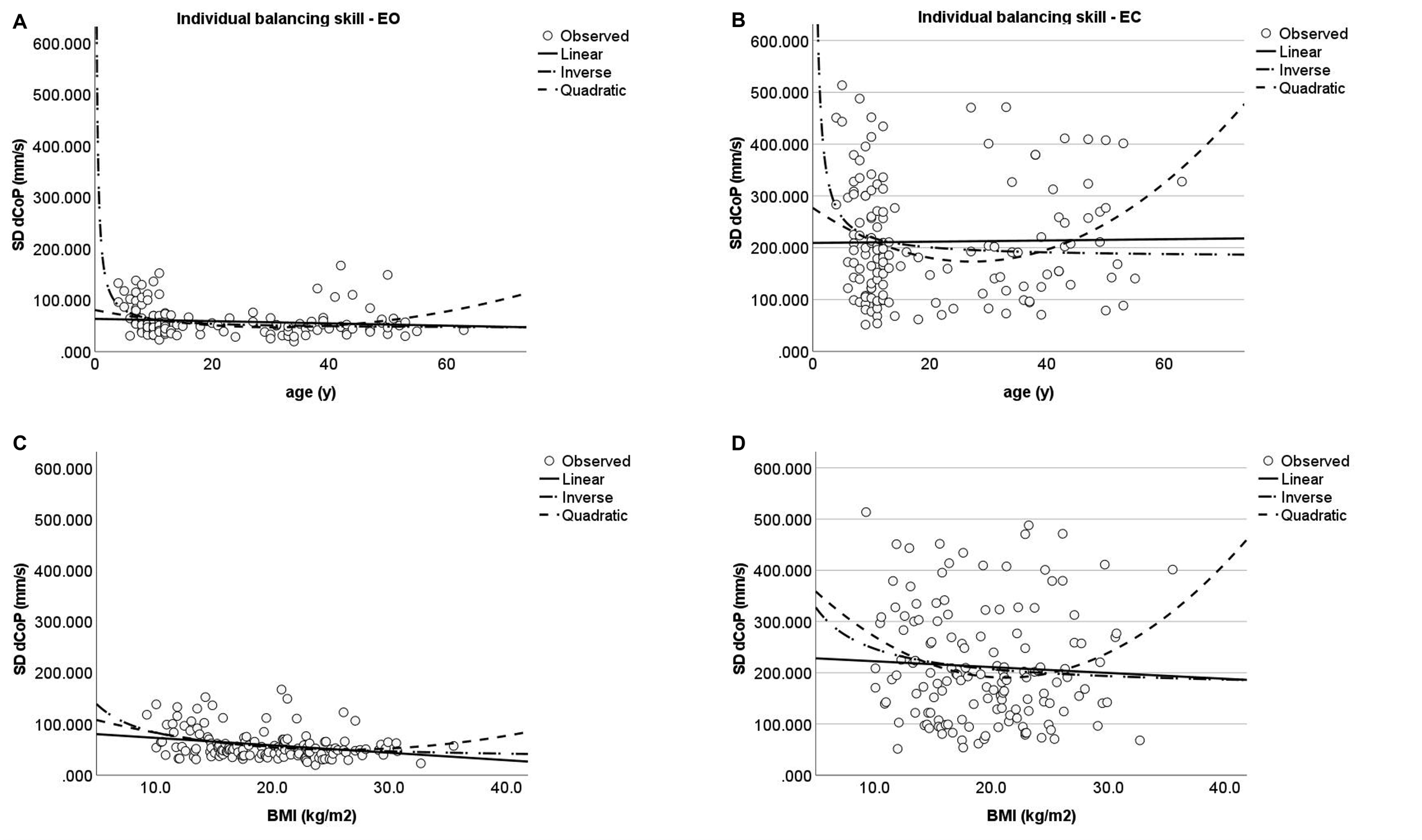

Supplement: S5 Fig — Curve fitting results of an individual’s balancing skill. With Eyes open (left) and Eyes closed(right) based on age-related motor experience (top) and BMI (bottom). (TIF) [file pone.0314946.s009.tif]

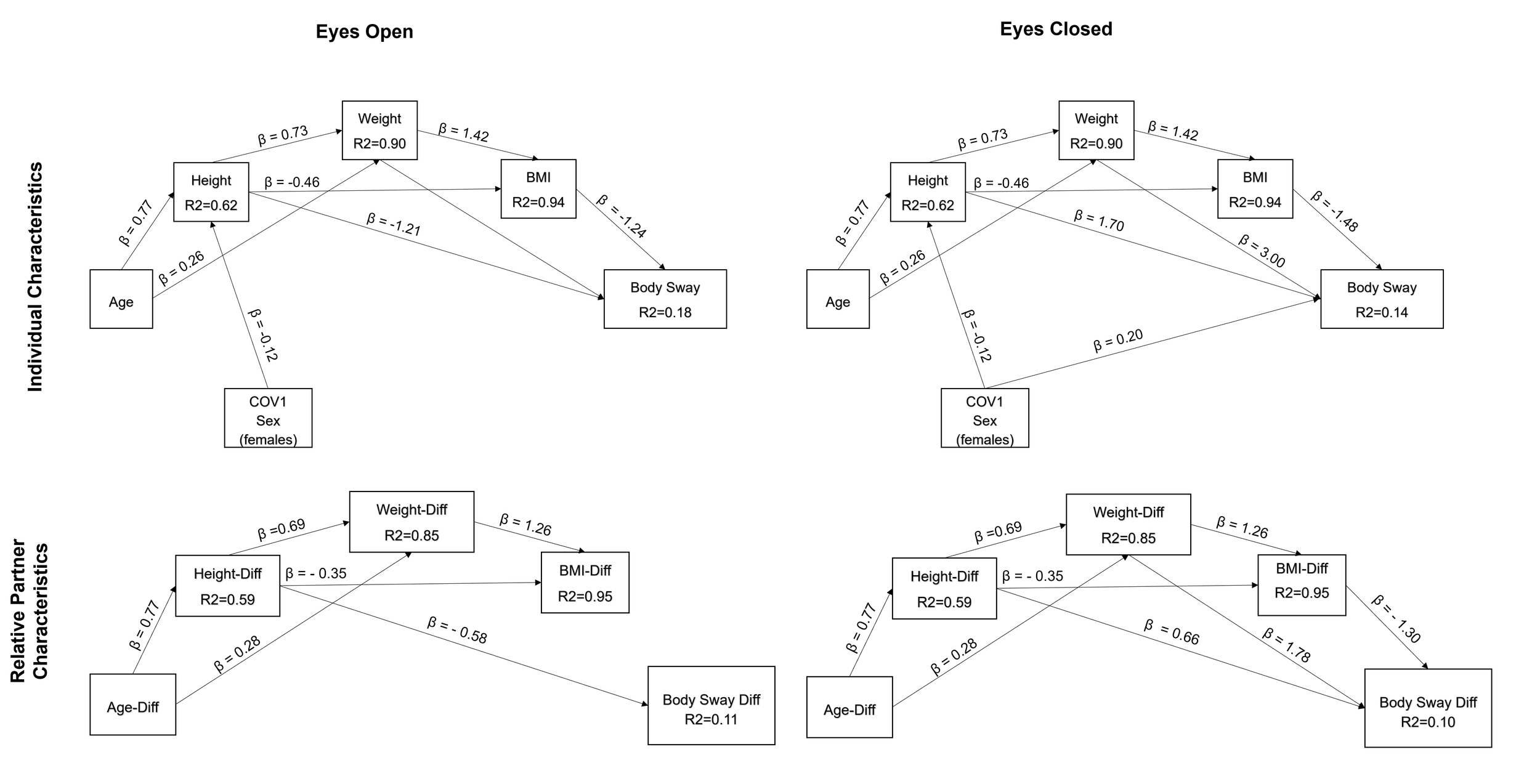

Supplement: S6 Fig — Statistical model of the influential factors on the benefit of IPT. (relative difference in variability in balancing skills with IPT compared to without IPT) for Eyes open condition (top) and Eyes closed condition (bottom). (TIF) [file pone.0314946.s010.tif]
