# Supplementary material for: The balance stabilising benefit of social touch: Influence of an individual’s age and the partner’s relative body characteristics
Source: PLoS One. 2025 Jun 5;20(6):e0314946. doi: 10.1371/journal.pone.0314946 (PMC12140250; doi:10.1371/journal.pone.0314946)
Supplement: S1 Appendix — Results description of a bootstrapped bivariate Pearson correlation analysis between individuals’ balancing skill and IPT benefit. (DOCX) [file pone.0314946.s011.docx]

**Supporting information and materials**

**S1 Appendix**.

The bootstrapped bivariate Pearson correlation analysis of the relative benefit of IPT across the whole group showed a moderate to high correlation with the interindividual differences in balancing skills (EO: r=-0.45, p<0.001, BCa95%CI [-0.58 -0.30]; EC: r=-0.54, p<0.001, BCa95%CI [-0.65 -0.40]). Bootstrapped bivariate Pearson correlations, did not reveal a significant relationship of an individual’s balancing skill (SD dCoP, no IPT) with age-related motor experience (r=-0.12, p=0.161, BCa95%CI [-0.28 0.05]), motor developmental potential (mean-centred age inverse) (r=-0.04, p=0.643, BCa95%CI [-0.11 0.03]) or extreme BMI (mean-centred BMI squared) (r=0.08, p=0.33, BCa95%CI [-0.07 0.27]) for Eyes open condition. Though, there was a significant low relationship of an individual’s balancing skill with height (r=-0.29, p<0.001, BCa95%CI [-0.45 -0.14]), weight (r=-0.25, p=0.002, BCa95%CI [-0.40 -12]) and BMI (r=-0.28, p<0.001, BCa95%CI [-0.42 -0.14]). Also, for the Eyes closed condition, age-related motor experience (r=0.02, p=0.847, BCa95%CI [-0.18 0.21]) and motor developmental potential (r=-0.06, p=0.505, BCa95%CI [-0.17 0.13]) were not significantly correlated with an individual’s balancing skill. In this condition only extreme BMI (r=0.18, p=0.031, BCa95%CI [-0.01 0.35]) showed a tendency for a low correlation, not however, height (r=-0.11, p=0.194, BCa95%CI [-0.29 0.09]), weight (r=-0.04, p=0.641, BCa95%CI [-0.23 0.15]) and BMI (r=-0.06, p=0.504, BCa95%CI [-0.25 0.15]).

However, the curve estimation for Eyes open condition showed the inverse relationship (R2=0.09, p<0.001) to be the best fit for describing the relationship an individual’s balancing skill with age-related motor experience compared to a linear relationship (R2=0.01, p=0.16), followed by the quadratic relationship (R2=0.06, p=0.012). For the relationship of BMI with body sway, the quadratic relationship (R2=0.11, p<0.001) better represented the relationship than the linear estimation (R2=0.08, p<0.001), though the linear relationship was also significant. For the relationship of an individual’s balancing skill and age-related motor experience during EC condition, the inverse (R2=0.03, p=0.045) and quadratic relationships (R2=0.05, p=0.029) revealed to be significant and to explain a greater amount of variance than the linear relationship (R2=0.000, p=0.847). The relationship of an individual’s balancing skill with BMI in the Eyes closed condition showed only the quadratic relationship (R2=0.05, p=0.040) to be significant and explained a greater amount of variance than the linear relationship (R2=0.003, p=0.504). To be consistent across sensory conditions and due to the low number of individuals aged >50 years (N=9) and >55 years (N=2), the inverse relationship was further used in the analysis for both Eyes open and Eyes closed.
