# Supplementary material for: The balance stabilising benefit of social touch: Influence of an individual’s age and the partner’s relative body characteristics
Source: PLoS One. 2025 Jun 5;20(6):e0314946. doi: 10.1371/journal.pone.0314946 (PMC12140250; doi:10.1371/journal.pone.0314946)
Supplement: S2 Appendix — Results description of serial regression analysis to predict an individual’s balancing skill. (DOCX) [file pone.0314946.s012.docx]

**Supporting information and materials**

**S2 Appendix.**

In the serial mediation analysis for the Eyes open condition, 18% of the variance of an individual’s balancing skill (SD dCoP) was explained by height, BMI and the full serial mediation of the effect of age via height, weight, and BMI. Greater body sway was explained by a lower body height (B=-174.61, BootMean=-169.49, BootSE=77.86, Boot95%CI [-304.31 -0.42]), β =-1.21, and lower BMI (B=-6.45, BootMean=-6.40, BootSE=2.24, Boot95%CI [-11.03 -1.80], β =-1.24). Age (B=0.56, BootMean=0.59, BootSE=0.36, Boot95%CI [-0.06 1.43], β =0.30), weight (B=2.14, BootMean=2.08, BootSE=1.11, Boot95%CI [-0.26 4.08], β =1.84), sex (B=-0.75, BootMean=-4.23, BootSE=5.12, Boot95%CI [-15.09 5.42], β =-0.07), age mean-centred inverse (B=-0.75, BootMean=-2.00, BootSE=6.25, Boot95%CI [-19.83 8.07], β =-0.01) and BMI mean-centred squared (B=0.10, BootMean=-0.10, BootSE=0.12, Boot95%CI [-0.34 0.15], β =-0.13) did not contribute significantly to the model. The effect of age on body sway was mediated via height (B=-1.75, BootSE=0.78, Boot95%CI [-3.05 -0.00], β =-0.93, BootSE=0.44, Boot95%CI [-1.74 -0.00]), height and BMI (B=0.83, BootSE=0.30, Boot95%CI [0.23 1.44], β =0.44, BootSE=0.17, Boot95%CI [0.12 0.78]), weight and BMI (B=-0.85, BootSE=0.38, Boot95%CI [-3.12 -0.50], β =-0.45, BootSE=0.21, Boot95%CI [-0.95 -0.11]), and via height, weight and BMI (B=-0.99, BootSE=0.64, Boot95%CI [-1.67 -0.26], β =-0.99, BootSE=0.35, Boot95%CI [-1.67 -0.26]).

For the Eyes closed condition, 14% of the variance of body sway was explained by height, weight, BMI and sex, as well as by the full serial mediation of the effect of age on body sway via height, weight, and BMI. Geater body sway was explained by a lower height (B=-955.55, BootMean=-934.89, BootSE=306.57, Boot95%CI [-1538.72 -346.46], β =-1.70), a greater weight (B=13.59, BootMean=13.35, BootSE=4.56, Boot95%CI [4.57 21.95], β =3.00), a lower BMI (B=-30.06, BootMean=-29.29, BootSE=10.63, Boot95%CI [-48.22 -6.77], β =-1.48) and for females (B=43.49, BootMean=43.54, BootSE=18.20, Boot95%CI [5.80 76.21], β =0.20). Age (B=0.73, BootMean=0.60, BootSE=1.25, Boot95%CI [-2.13 2.76], β =0.10), age mean-centred inverse (B=-20.52, BootMean=-20.45, BootSE=43.02, Boot95%CI[-118.82 76.80], β =-0.06), and BMI mean-centred squared (B=-0.73, BootMean=-0.74, BootSE=0.50, Boot95%CI [-1.75 0.21], β =-0.24) did not contribute significantly to the model. However, the effect of age was mediated via height (B=-9.59, BootSE=3.21, Boot95%CI [-15.61 -3.35], β =-1.31, BootSE=0.40, Boot95%CI [-2.04 -0.48]), weight (B=5.66, BootSE=2.31, Boot95%CI [1.53 10.63], β =0.78, BootSE=0.30, Boot95%CI [0.22 1.39]), via height and weight (B=12.30, BootSE=4.46, Boot95%CI [3.81 21.35]), via height and BMI (B=3.87, BootSE=1.45, Boot95% CI [0.83 6.67], β =0.53, BootSE=0.19, Boot95%CI [0.12, 0.89]), via weight and BMI (B=-3.97, BootSE=1.61, Boot95%CI [-7.24 -0.91], β =-0.54, BootSE=0.21, Boot95%CI [-0.95 -0.13]), and via height, weight and BMI (B=-8.61, Boot 95%CI [-14.97 -1.80], β =-1.18, BootSE=0.42, Boot95%CI [-1.98 -0.26]).

Moreover, the variance of interindividual differences in balancing skills during Eyes open condition was explained to 11% by height difference and a mediated effect of age difference via height difference. A more positive interindividual difference in balancing skill was explained by a lower height difference (B=-1.83, BootMean=-1.77, BootSE=0.61, Boot 95%CI [-2.91 -0.50], β =-0.58) and the mediated effect of age difference via height difference (B=-0.02, BootSE=0.01, Boot95%CI [-0.03 -0.01], β =-0.44, BootSE=0.17, Boot95%CI [-0.76 -0.10]). Age difference (B=0.01, BootMean=0.01, BootSE=0.01, Boot95%CI [-0.00 0.03], β =0.30), weight difference (B=0.02, BootMean=0.01, BootSE=0.01, Boot95%CI [-0.00 0.03], β =0.66), BMI difference (B=-0.06, BootMean=-0.06, BootSE=0.04, Boot95%CI [-0.13 0.02], β =-0.60) and sex difference (B=-0.03, BootMean=-0.03, BootSE=0.12, Boot 95%CI [-0.25 0.19], β =-0.02) did not have a significant direct effect.

In the Eyes closed condition, interindividual differences in balancing skills were explained to 10% by height difference (B=-2.60, BootMean=-2.53, BootSE=0.81, Boot95%CI [-4.28 -1.02], β =-0.66), weight difference (B=0.05, BootMean=0.05, BootSE=0.02, Boot95%CI [0.02 0.08], β =1.78), and BMI difference (B=-0.16, BootMean=-0.16, BootSE=0.05, Boot95%CI [-0.25 -0.05], β =-1.30). Moreover, differently to the Eyes open condition, during Eyes open the effect of age difference on interindividual differences in balancing skills was fully mediated via height-, weight- and BMI differences (B=0.04, Boot95%CI [-0.07 -0.01], β =-0.87, BootSE=0.31, Boot95%CI [-1.39 -0.29]), in addition to via height difference only (B=-0.02, BootSE=0.01, Boot95%CI [-0.04 -0.01], β =-0.50, BootSE=0.16, Boot95%CI [-0.84 -0.20]), via weight difference (B=0.02, BootSE=0.01, Boot95%CI [0.01, 0.04], β =0.50, BootSE=0.18, Boot95%CI [0.15 0.87]), via height and weight differences (B=0.04, BootSE=0.02, Boot95%CI [0.02 0.08], β =0.94, BootSE=0.31, Boot95%CI [0.32, 1.55]), via height and BMI differences (B=-0.02, BootSE=0.01, Boot95%CI [0.01, 0.03], β =-0.35, BootSE=0.12, Boot95%CI [0.10 0.57]), and via weight and BMI differences (B=-0.02, BootSE=0.01, Boot95%CI [-0.04, -0.01], β =-0.46, BootSE=0.16, Boot95%CI [-0.77 -0.14]). Age difference (B=0.00, BootMean=0.00, BootSE=0.01, Boot95%CI [-0.01 0.01], β =0.02), and sex difference (B=-0.01, BootMean=-0.01, BootSE=0.14, Boot95%CI [-0.29 0.29], β =-0.00) did again not show any significant direct effect.
